# Predicting biomedical relationships using the knowledge and graph embedding cascade model

**DOI:** 10.1371/journal.pone.0218264

**Published:** 2019-06-13

**Authors:** Xiaomin Liang, Daifeng Li, Min Song, Andrew Madden, Ying Ding, Yi Bu

**Affiliations:** 1 School of Information Management, Sun Yat-Sen Uniersity, Guangzhou, Guangdong, China; 2 Department of Library and Information Science, Yonsei University, Seoul, Korea; 3 School of Informatics, Computing, and Engineering, Indiana University, Bloomington, Indiana, United States of America; 4 School of Information Management, Wuhan University, Wuhan, Hubei, China; Northwestern Polytechnical University, CHINA

## Abstract

Advances in machine learning and deep learning methods, together with the increasing availability of large-scale pharmacological, genomic, and chemical datasets, have created opportunities for identifying potentially useful relationships within biochemical networks. Knowledge embedding models have been found to have value in detecting knowledge-based correlations among entities, but little effort has been made to apply them to networks of biochemical entities. This is because such networks tend to be unbalanced and sparse, and knowledge embedding models do not work well on them. However, to some extent, the shortcomings of knowledge embedding models can be compensated for if they are used in association with graph embedding. In this paper, we combine knowledge embedding and graph embedding to represent biochemical entities and their relations as dense and low-dimensional vectors. We build a cascade learning framework which incorporates semantic features from the knowledge embedding model, and graph features from the graph embedding model, to score the probability of linking. The proposed method performs noticeably better than the models with which it is compared. It predicted links and entities with an accuracy of 93%, and its average hits@10 score has an average of 8.6% absolute improvement compared with original knowledge embedding model, 1.1% to 9.7% absolute improvement compared with other knowledge and graph embedding algorithm. In addition, we designed a meta-path algorithm to detect path relations in the biomedical network. Case studies further verify the value of the proposed model in finding potential relationships between diseases, drugs, genes, treatments, etc. Amongst the findings of the proposed model are the suggestion that VDR (vitamin D receptor) may be linked to prostate cancer. This is backed by evidence from medical databases and published research, supporting the suggestion that our proposed model could be of value to biomedical researchers.

## Introduction

Biochemistry is a cross-discipline, incorporating elements of pharmacology, biology, and chemistry. The large number of disciplines associated with biochemistry makes it challenge to identify new relationships. Computational prediction is becoming a crucial and effective strategy for identifying links, given its potentials to reduce the high failure risk of expensive and time-consuming laboratory experiments.

Traditional computational methods are mainly based on molecular docking [1] and ligand chemistry [2], but more efforts have been put into network-based approaches in the past decade. Using these approaches, the prediction problem is equivalent to link prediction, which is a fundamental and crucial problem in complex network analytics. Network-based approaches are based on assumption of “guilt-by association”—for example, similar drugs may interact with similar genes and *vice versa*. Such similarities are calculated from direct connections or from common neighbors, which are, therefore, limited to bipartite networks [3, 4]. In order to improve the accuracy of predictions, researchers have tried many strategies, including the incorporation of new contexts and the usage of new types of data (e.g., genomic data, drug-disease interaction data, side-effects, etc. [5–7]). Despite of these, biochemical networks are becoming large-scale, complex, and heterogeneous, with multiple relations making a homogeneous algorithm unsuitable for biochemical network inferences. Recently, several computational methods (such as resource diffusion [8, 9], and random walk [10, 11], have been proposed to integrate heterogeneous data to predict potential links. In addition, meta-path-based network analysis has been developed to compute topological features like path counts and random walk [12, 13]. Luo *et al* [14] for instance, applied a compact feature learning algorithm to obtain the topological properties for each node. They considered that networks can be represented by matrices: similarity matrixes are computed using operations such as matrix multiplication and matrix factorization. Another relevant research branch made use of information lying in biochemical text, such as biochemical research literature and clinical reports. Some hypothesis generation systems are constructed using topic models together with biochemical triplets [15, 16]. Word embedding is introduced to represent biochemical entities and then to measure the similarities between them [17, 18].

Unlike earlier studies, we attempt to use knowledge graph-based representation methods to solve the problem of link prediction and entity prediction. Knowledge graph representation models (Knowledge embedding) are popular in recent years, especially TransX series [19–22], because of their efficiency and effectiveness; but there have been few attempts to apply them to the area of biochemistry. This is due to the fact that biochemical data are always unbalanced and sparse, which greatly limits the performance of knowledge graph embedding models. To address this problem, the proposed framework incorporates graph topological features (graph embedding) to make up the limitations, and it works out fine according to the experiment results.

Knowledge graphs are graph-structured knowledge bases, in which facts are represented as relations (edges) between entities (nodes). Resource Description Framework (RDF) is a common way of representing knowledge graphs. RDF defines relationships in the form of triplets comprising head entity, relation, and tail entity (denoted as (h, r, t) in the present paper). Biochemical networks can be treated as knowledge graphs, where vertexes are the entities, and edges are the relations from head entity to tail entity. Thus, a link prediction task in biochemical networks is equivalent to a knowledge graph completion task. In recent research studies, the methodology of knowledge embedding has been popular for drawing inferences based on knowledge graphs. The main advantage of knowledge embedding is that it maps concepts and relations into dense, low-dimensional vectors, so relations between various entities can be described using different mathematical methods, further improving the semantic understanding of knowledge graphs. Among popular and efficient tools of knowledge embedding is translation-based models. They map the head (h) and tail (t) into a low-dimensional vector space and treat the relation (r) as a translation from h to t for the triplet (h, r, t). The translation principle is defined as: *h* + *r* ≈ *t*, so the score of a triplet can be defined as the distance between (h + r) and (t): *f* = ‖*h* + *r* − *t*‖. If (h, r, t) holds, (t) should be close to (h + r) in embedding space, otherwise, (h + r) should be far away from t [19]. Within the translation-based models, there are some variant models that employ different mapping strategies to map entities into embedding space while trying to retain as much information as possible from the knowledge graphs, including TransE [19], TransH [20], TransR [21], TranSparse [22], etc.

**Fig 1:**
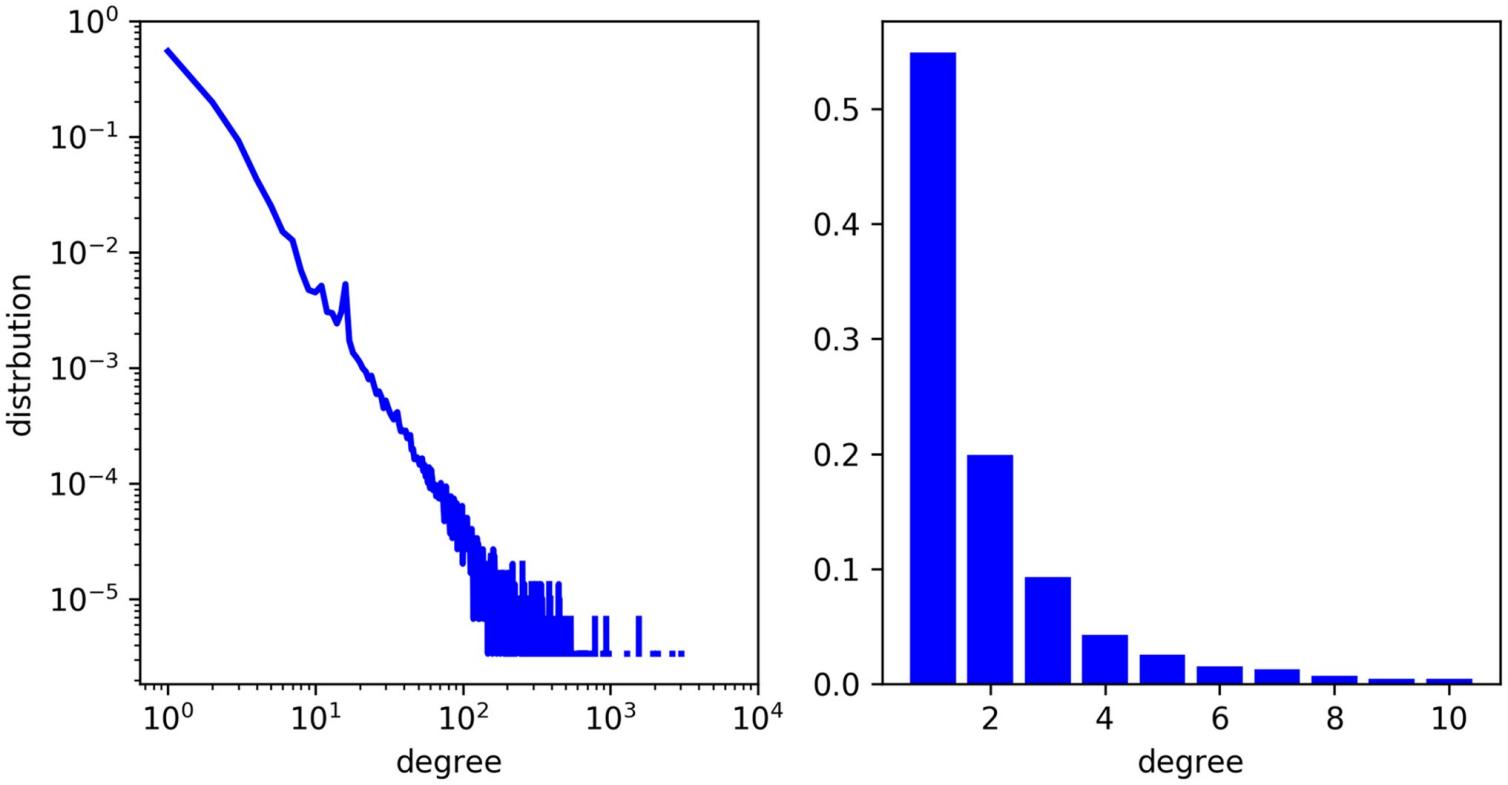
Degree distribution of Chem2Bio2Rdf.

Translation-based embedding models have performed well on their experimental data sets, and there have been a few studies that apply them to biochemical link prediction (eg, [23–25]. However, translation-based models are not always effective if applied directly to biochemical data. One reason is that there are a limited number of links for each entity on average, constraining the information available for learning *h* + *r* ≈ *t* patterns. Chem2Bio2RDF [26], for example, is a set of complex heterogeneous network records with around 720,000 relations and 296,000 entities. The average degree is around five, but actually more than half nodes’ degrees are just one, and around 80% nodes’ degrees are not more than three (see Fig 1. In addition, biochemical networks often contain unbalanced relationships, e.g. one-to-many, many-to-one, and many-to-many. This creates a challenge for translation-based embedding models since there are not enough contexts to map n-side entities to suitable positions in the embedding space.

To solve this problem, we treat the knowledge graph as not only a set of triplets but also a graph. Graph features (such as hemophilic and structural equivalences) are also important elements of knowledge graphs, so we propose a model that introduces graph features to help with link predictions. Graph embedding models allow topological features to be represented.

Many graph embedding methods have been proposed in recent years in the broader field of representation learning, leading to significant progress in automating prediction. However, present graph embedding approaches do not capture the diversity of connectivity patterns observed in networks [27]. Node2vec was chosen to capture graph features instead of other methods for reasons given below. Three of the most representative graph embedding methods are used as examples, i.e.: DeepWalk, LINE and SDNE.

DeepWalk [28] learns d-dimensional feature representations by simulating uniform random walks. The sampling strategy used in DeepWalk can be seen as a special case of node2vec with p = 1 and q = 1.

LINE [29] learns d-dimensional feature representations in two separate phases. In the first phase, it learns d = 2 dimensions by BFS-style (Breath First Search) simulations over immediate neighbors of nodes. In the second phase, it learns the next d = 2 dimensions by sampling nodes strictly at a 2-hop distance from the source nodes.

SDNE [30] is a semi-supervised deep model, which can exploit first-order and second-order proximity jointly to preserve the network structure. The method can preserve both the local and global network structure and is robust on sparse networks. Highly non-linear network structures result in sub-optimal network representations: the strategy adopted by SDNE helps to address this problem.

According to [27], Node2vec outperforms both DeepWalk and LINE on three different network datasets. Node2vec’s main innovation is to improve on the strategy of an optimized random walk. It defines two parameters, p and q, which strike a balance between BFS and DFS; it also considers both local and global information, which makes it more adaptive towards different networks. One of the disadvantages of LINE is its inability to reuse samples. Node2vec’s usage of random walk methods allows it to avoid this shortcoming. Node2vec also improves on SDNE by being more efficient and through its ability to handle large-scale networks (node2vec needs 4 hours to run a network with 1 million triples). In another aspect, SDNE is more suitable for network with highly non-linear attributes, such as Filckr and Youtube, for biomedical network, the relations among entities are relatively stable, so Node2vec should be an optimal choice. The experimental results presented in Section “Experiment and results” further verify our analysis.

**Fig 2:**
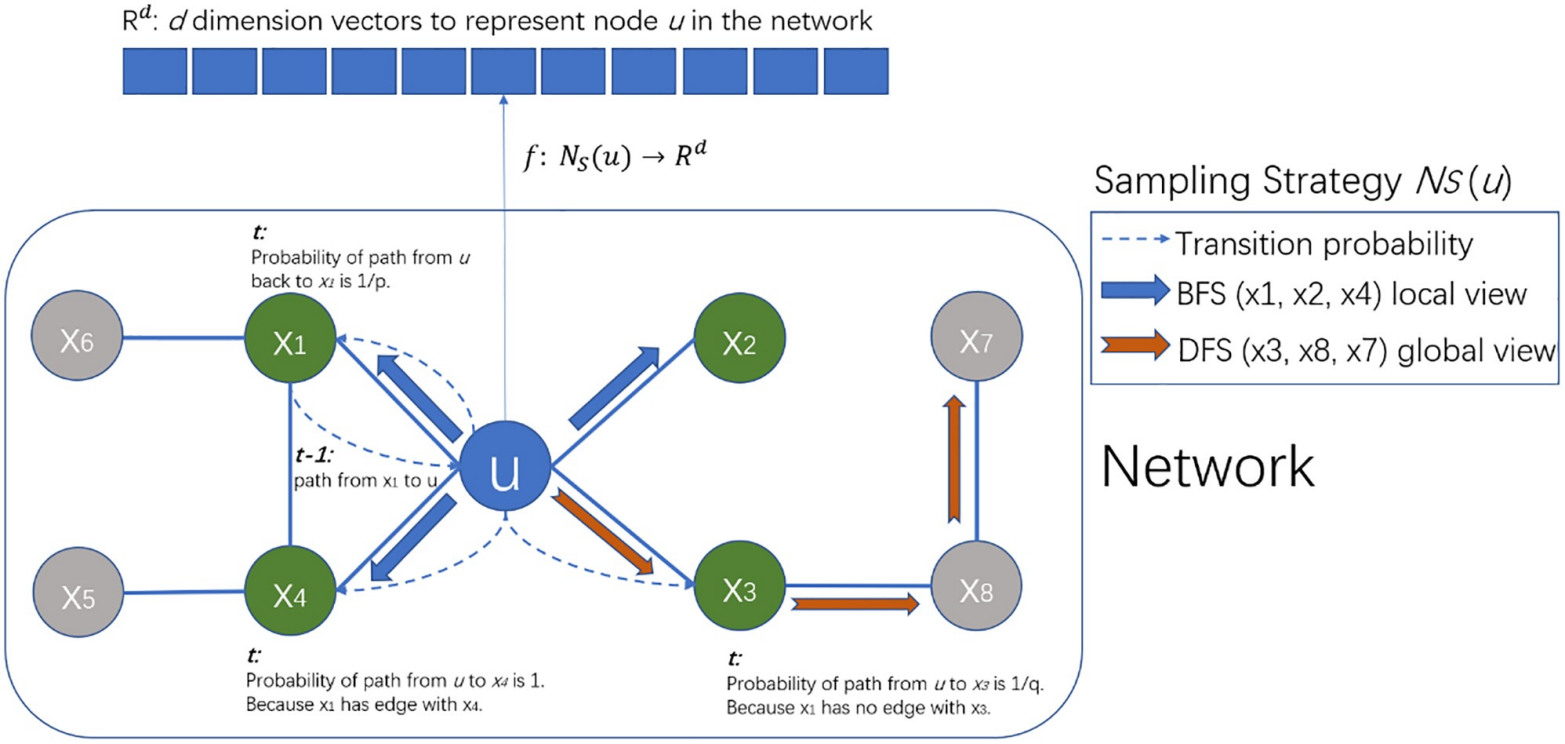
The framework of Node2vec. Developed from Node2vec [27].

For these reasons, we chose to use Node2vec to generate graph features. Both Node2vec and Word2vec [31, 32] (from which Node2vec was developed) are skip-gram models. In word2vec, the input is one word and the output contains words which surround the input. In node2vec the input is one node, and the output is surrounding nodes. The problem is how to define the surrounding nodes. Node2vec conducts random walks for each node combining two classic search strategies: breadth first strategy (BFS) and depth first strategy (DFS), to encode local context and global context. For example, node u in Fig 2 has surroundings X1, X2, X4 if a BFS strategy is used, and X3, X8, X7 if a DFS strategy is used. Node2vec defines two parameters (p and q) to control the balance between BFS and DFS. Assuming there is a path from X1 to u at time t -1, then at time t, the return probability from u to X1 is 1/p. The probability of transition from u to X4 should be 1 because an edge exists between X1 and X4. The transition probability from u to X2 and X3 is 1/q because there are no edges between them and X1. Node2vec provides flexibility in combining BFS and DFS by adjusting hyper-parameters.

To make the best use of knowledge embedding and graph embedding, we propose using a cascade learning framework that incorporates both of them to predict potential links in biochemical networks. Cascade learning is a strategy that uses a sequence of functions to approach the true value. It estimates the importance of a feature stage by stage, and eliminates noisy features or items. Cascade learning was first applied to tasks that involve visual object detection [33, 34], and was subsequently applied to ranking [35–37]. Knowledge embedding and graph embedding can be regarded as a rough expression of entity relations. The proposed cascade model fuses and refines feature expression, and finally produces an optimized result.

Our contributions are: 1) a translation-based knowledge graph embedding model is leveraged to handle biochemical link prediction, which is efficient, effective, and suitable for large biochemical data; 2) graph features are introduced to address the poor performance of translation-based embedding models on unbalanced relationships; we use graph embedding model to encode local and global graph features; 3) a cascade learning framework is proposed in this work to incorporate knowledge embedding features and graph features, The framework takes advantages of both sets of features and achieve obvious improvements; 4) we comprehensively evaluate the performance of the proposed method in link prediction, entity prediction and path predictions. Some example cases are provided to demonstrate the application of the proposed model.

## Materials and methods

### Data

The dataset BioChem used in this work consists of around 720,000 RDF triplets, which are derived from Chem2bio2RDF [26]. Twelve distinct relations are involved in the dataset, including *Has chemical ontology*, *Bind*, *Express*, *Has gene gamily*, *Protein-Protein interaction*, *Expressed in*, *Has participants*, *Treated by*, *Caused by*, Induced by, *Has substructure*, *Has gene ontology*. The subject entity type, object entity type, the numbers of entity type and the count of triplets in each relation are reported in Table 1.

**Table 1:** The statistics of dataset Chem2Bio2Rdf.

Relation index	Relation	Relation description	Head entity type	tail entity type	Number of triplets
R1	CHEBI	has chemical ontology	compound	chemical ontology	14407
R2	chemogenomic	bind	compound	gene	515865
R3	expression	express	compound	gene	15884
R4	family_name	has gene family	gene family	gene	7112
R5	hprd	protein-protein interaction	gene	gene	29677
R6	tissue	expressed in	tissue	gene	9730
R7	protein	has pathwat	pathway	gene	10583
R8	drug	treated by	disease	compound	909
R9	gene	caused by	disease	gene	2646
R10	cid	induced by	side effect	compound	8852
R11	substructure	has substructure	compound	substructure	6030
R12	GO_id	has gene ontology	gene	gene ontology	15884

Total triplets: 719865; Total entities: 295911

### TranSparse

TranSparse [22] is a variant model that maps entities and relations in distinct space. The entities are projected into a relation-specific space via a distinct sparse project matrix, which is low-rank and more flexible. The score function of TranSparse is:

$$\begin{array}{c} d\left(h,r,t\right)=\left|\right|h_{p}+r-t_{p}{\left|\right|}_{L1/2} \end{array}$$ (1)

Where

$$\begin{array}{c} h_{p}=M{}_{r}^{h}\left(\theta {}_{r}^{h}\right)h,t_{p}=M{}_{r}^{t}\left(\theta {}_{r}^{t}\right)t \end{array}$$ (2)

h, t are n-dimensional vectors representing head and tail entities. $M{}_{r}^{h}$ and $M{}_{r}^{t}$ are *m* × *n* matrices used to project h and t into a m dimensional space related to relation r. The proportion of zeros in a matrix is called the sparse degree, and $\theta {}_{r}^{h}$ and $\theta {}_{r}^{t}$ are the sparse degrees of transfer matrices related to h and t. TranSparse is a margin-based model, the objective of which is to minimize the loss function defined as follows:

$$\begin{array}{c} \min L=\sum_{(h,r,t)\in \Delta }\sum_{\left(h^{\prime },r,t^{\prime }\right)h\in \Delta^{\prime }(h,r,t)}[\gamma +d\left(h,r,t\right)-d\left(h^{\prime },r,t^{\prime }\right){]}_{+} \end{array}$$ (3)

where ${\left[x\right]}_{+}\;≜\;max\left(0,x\right),\gamma >0$ is a margin hyperparameter, Δ is the set of positive triplets, and Δ′ is the set of negative triplets generated by randomly replacing a head entity or a tail entity. Loss L is global loss involving all entities and relations; consequently the embedding of entities or relations contains global information from the whole knowledge graph.

### Node2vec

After processing all entities with a random walk based on a combination of BFS and DFS (see Fig 2), we obtain a set of neighbor sequences for each entity.

Let G = 〈*V*, *R*〉 be a given network. For each entity *u* ∈ *V*, its neighbor entity sequences are defined as N(u). The representation vectors are learned through the mapping function $f:u\to R^{d}$. The objective function, which maximizes the logarithmic probability of observing neighborhood N(u) given the feature representation of entity u, is defined as follows:

$$\begin{array}{c} \max_{f}=\sum_{u\in V}\log\left(P\left(N\left(u\right)\right|f\left(u\right)\right) \end{array}$$ (4)

This ensures that the entity with similar neighbors will be close by in a feature space.

An edge function g(u,v) is defined to measure the structural similarity of two entities, u and v. g(u, v) is a binary operator (such as average, hadamard, L1-norm, or L2-norm) between the corresponding vectors f(u) and f(v). Hadamard production has been proved best in Node2vec [27].

**Fig 3:**
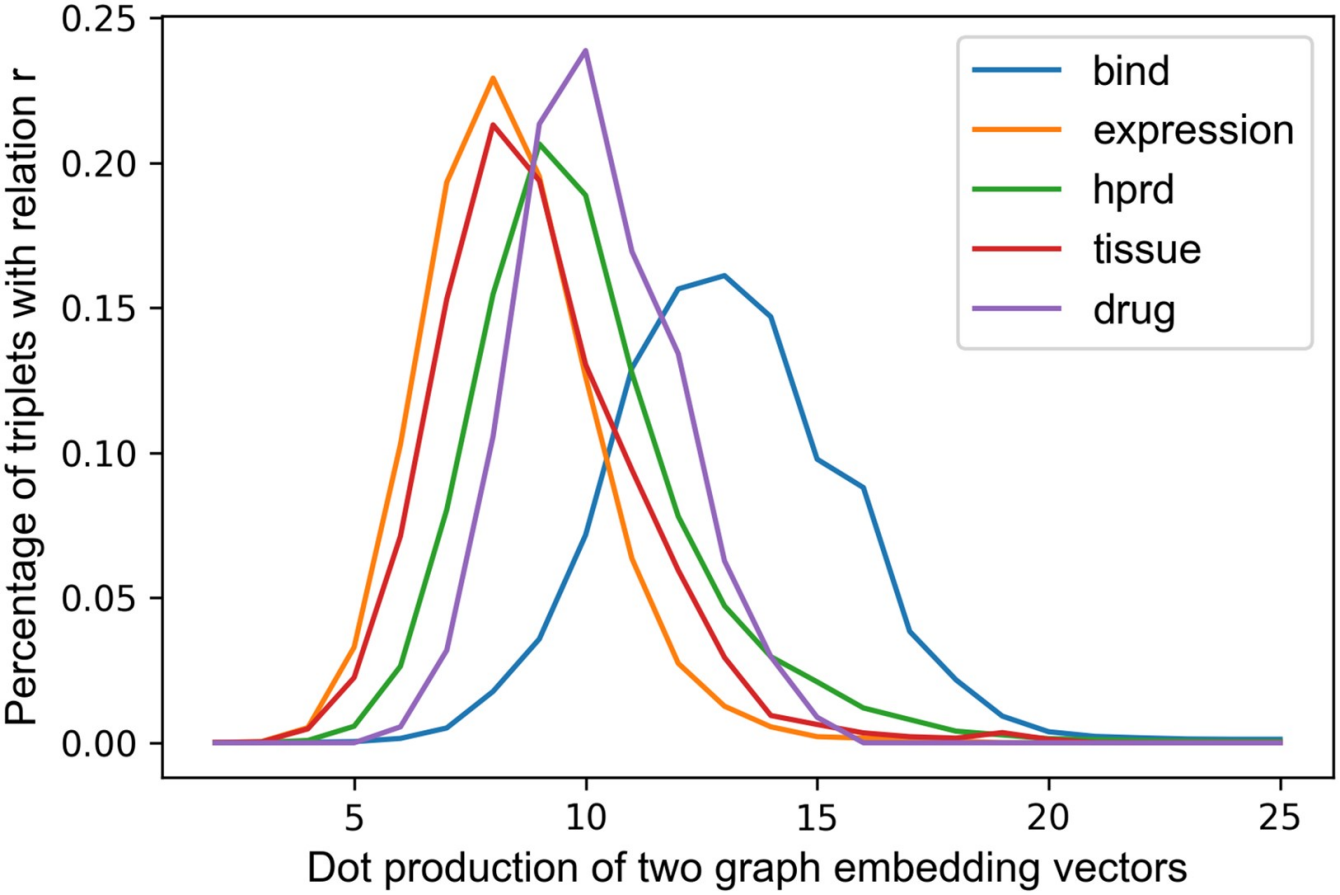
Correlation between graph embedding and linking.

Node2vec is designed for homogeneous networks, however biochemical network Chem2Bio2Rdf are heterogeneous and multi-relational. But we find that there is at most one relation between different entity pairs in Chem2Bio2Rdf, so we treat it as a homogeneous network of one relationship (linked or not linked) and feed it into Node2vec to get the representations of entities in the network. We assume that the graph embedding distance of two entities is positively correlated to their specific relation r. To test this, we select five relations and draw the distribution of their Node2vec dot production distance under certain relation types. The distribution (Fig 3) shows that the distance between two entities correlates to their relation types. However different relations have different distribution shapes; for example, the dot production distance of most node pairs with relation bind are from 10 to 17, the distance value of 16% node pairs with relation bind is around 13.5. Fig 3 supports our assumption and shows that Node2vec is helpful for link prediction. So the remaining problem is how to extract the correlation between graph embedding distances and relations.

### Cascade learning framework

To make the best use of knowledge embedding and graph embedding as aforementioned, a cascade learning framework is used to combine them into a unified framework. In the present paper, the graph of biomedical domain is in the form of triplets (*h*, *r*, *t* ∈ *T*) comprising head and tail entities (h,t) which are the members of E (the set of entities). Given this, and the relationship r ∈ R (the set of relationships) to indicate the relation from the head to the tail, the knowledge embedding and graph embedding distance can be seen as feature descriptions of entities and relations. For each triplet (h, r, t), knowledge embedding feature F is defined as: *F*(*h*, *t*) = |*h_r_* + *r* − *t_r_*|, where *h*_*r*_*andt*_*r*_ are the projected vectors of head h and tail t entities on relation r. Graph embedding feature G is defined as G(h, t) = u(h) * u(t), where u(h) and u(t) are graph embedding of h and t respectively, * means hadamard product. So the original feature set is [*F*, *G*]. The negative generation protocol in this model follows the translation-based model [19]: negative triplet set Δ′ comprises training triplets with either the head or tail randomly replaced by a candidate entity.

$$\begin{array}{c} \Delta_{\left(h,r,t\right)}^{\prime }=\{\left(h^{\prime },r,t\right)|h^{\prime }\in E\}\cup \{\left(h,r,t^{\prime }\right)|t^{\prime }\in E\} \end{array}$$ (5)

[*F*, *G*] provides the initial inputs for the cascade model, which then optimizes them stage by stage. Furthermore, it can detect and make use of additional information from [F, G] for more accurate inference of relations.

**Fig 4:**
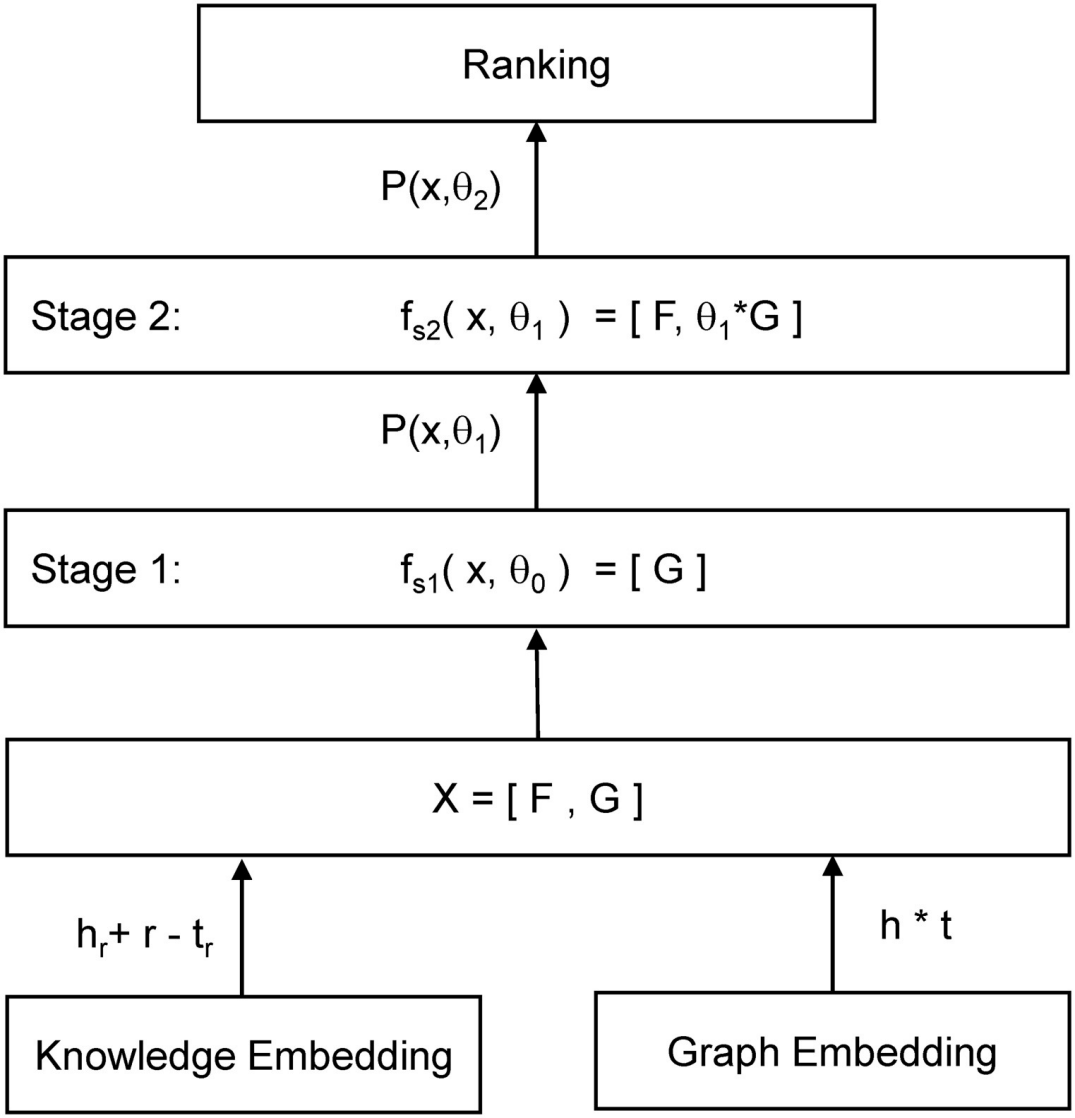
The framework of knowledge embedding cascade model.

Let [*S*_1_, *S*_2_, …, *S*_*T*_] denotes a T stage cascade, where each stage *S*_*i*_ is an independent classifier using a subset x of all features *X* = {*x*_*k*_, *k* = 1, 2, …, *n*}. *f*_*s*,*i*_ is a feature select operation in the i-th stage with estimated parameters *θ*_*i*−1_ learned from the (i-1)-th stage. A two-stage cascade learning model is constructed in this paper since there are only two kinds of feature [*F*, *G*] (see Fig 4). A logistic sigmoid function is used for a single stage classifier.

$$\begin{array}{c} P\left(x,\theta_{x,i}\right)=\sigma \left(\theta {}_{x,i}^{T}f_{s,i}\left(x,\theta_{i-1}\right)\right) \end{array}$$ (6)

where *P*(*x*, *θ*_*i*_) is the probability of x predicted as positive in the i-th stage. *σ*(*z*) = 1/(1 + *exp*(−*z*)) is the standard sigmoid function. The probability of the j-th instance (h, r, t) in the training set (Δ ∪ Δ′) being positive is defined as the probability of final stage n:

$$\begin{array}{c} P_{j}\left(y_{j}=1\right)=P\left(x_{j},\theta_{n}\right) \end{array}$$ (7)

Thus the log-likelihood function is:

$$\begin{array}{c} L\left(\theta \right)=-[\sum_{\left(h,r,t\right)\in \left(\Delta \cup \Delta^{\prime }\right)}y_{i}\log p_{i}+\left(1-y_{i}\right)\log(1-p\left(y_{i}\right)]+\alpha {∥\theta ∥}_{2} \end{array}$$ (8)

*α*‖*θ*‖_2_ is L2-norm regulation to address multiple collinearity and overfitting. In order to correlate graph features with the final objective, graph features go through stage one alone and then concatenate with knowledge embedding in stage two. The final ranking is based on the *P*(*x*, *θ*) at the final stage.

### Applications

Performance of the proposed model and other baselines are evaluated by predicting whether testing triplets hold. We consider three tasks: link prediction, entity prediction and path prediction.

#### Link prediction

Given a biochemical triplet (h, r, t), we can calculate the probability p that this triplet is True, and set a threshold *δ*. If *p* > *δ*, then the triplet is predicted to be True, otherwise it is predicted to be False.

#### Entity prediction

We can find potential head or tail entities for specific entities and relations. Given head entity h and relation r (h, r, ?), to predict tail entity t; or given tail entity t and relation r (?, r, t), to predict head entity h. For example, to help drug discovery, the model can return potential drugs for a specific gene, or potential genes for a specific drug.

#### Path prediction

**Fig 5:**
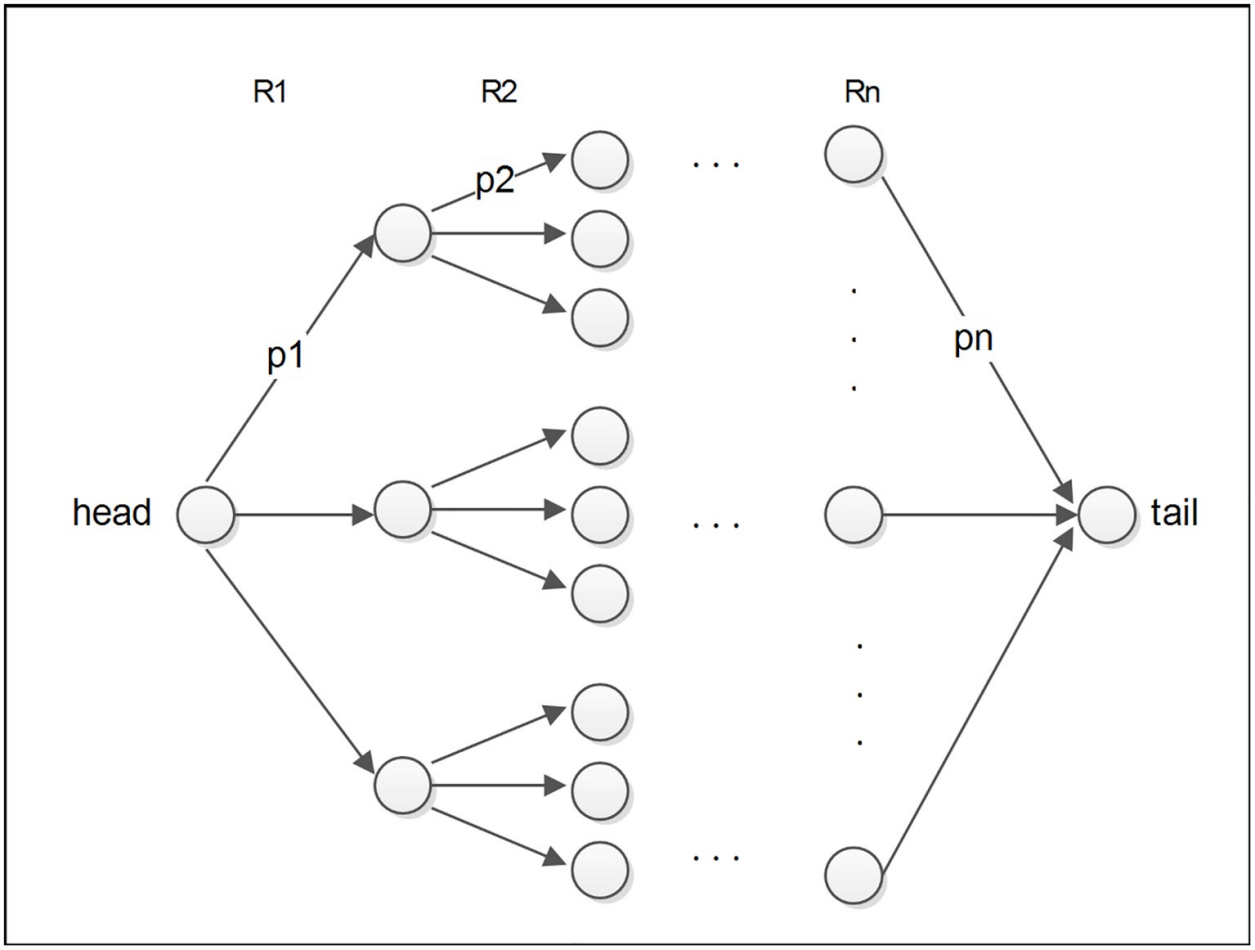
Path prediction for a specific head and tail.

The paths in which two entities interact with each other is of great interest in biochemistry. We incorporate meta-paths to predict optimal paths connecting two specific entities using a greedy algorithm (see Fig 5). Given a head entity h and a tail entity t, in potential reaction paths between h and t *h*, *m*_*i*_, *t*, *m*_*i*_ are the intermediate reactants between h and t along the meta-path p consisting of *R*_1_/*R*_2_/…/*R_n_*. The process of calculating path reliability is illustrated in Fig 5. For the ith relation *R*_*i*_ along the meta-path, assuming its head entity is *h*_*i*_, we extract the top k tail entities, which will be the head candidates for the next relation *R*_*i*+1_. The reliability of path is the production of link probability calculated by the cascade model: P(path) = *p*_*R*1_ × *p*_*R*2_ × … ×*p*_*Rn*_ × *p*_*ht*_. Here, *p*_*Ri*_ denotes the link probability in each relation along meta-path, and pht denotes the link probability of head entity and tail entity (h, r, t). Taking compound-disease-gene meta-path as an example, we can calculate the probability of relation bind between a compound and a gene that are both related to a given disease. This could be helpful in drug repositioning.

## Experiment and results

### Experiment set up

In this experiment, we used as a testing set, 10k triplets extracted from a biochemical triplet set; the remainder served as a training set. We ran TranSparse (http://www.nlpr.ia.ac.cn/cip/liukang/liukangPageFile/code/TransSparse.rar) and Node2vec (https://github.com/aditya-grover/node2vec) on the training set using the code provided by their authors. Optimized parameter assignment in TranSparse was *γ* = 1.5, *α* = 0.001, *k* = 100, *epochs* = 1000, where *γ* is the margin between positive triplet and negative triplet, *α* is the learning rate, k is the dimension of embedding vectors and and L1 is dissimilarity. In Node2vec, p = 1, q = 1, k = 128, length of walks = 80, context size = 10, number of walks per node = 3, where p and q are parameters controlling the random walk, and k is the dimension of embedding vectors. The annotation of these parameters and the sensitivity of the proposed cascade model to parameters of Node2vec are represented in the section “entity prediction”. In this experiment, some other knowledge embedding models and graph embedding models are taken as baselines. For TransH and TransR, the margin is set as 1, the learning rate is 0.001, and the dimension of representation vectors is set to 100, the number of iteration is set to 1000. For LINE, the number of negative samples is set as 5 and the total number of samples is 10 billion, the dimension of the embedding is set as 128 and the learning rate is 0.025. For SDNE, the hyper parameter *α* is set as 100, *γ* is 1, and *β* is 50; The batch size is set as 32, the learning rate is 0.01 and the epochs is 20; The dbn is set as True, and the batch size of dbn is set as 64, the learning rate is 0.1 and the epochs of dbn is 20.

Three tasks were set up in our experiment. These correspond to the three applications of the proposed method discussed in the section “Applications”, i.e. link prediction, entity prediction, and path prediction.

### Link prediction

Link prediction is a binary classification task in which a given triplet (h, r, t) is judged to be correct or not. The testing set only contains correct triplets, so we constructed negative testing triplets in the same way as we generated negative training triplets: by randomly replacing head or tail entities with other entities for each test triplet (see Eq 5).

We use accuracy to evaluate the performance of triplet classification. For TranSparse, we set a threshold *δ*_*r*_ for each relation r. *δ*_*r*_ is obtained by maximizing the classification accuracy on training set. For a given triplet (h, r, t), if its dissimilarity score is lower than *δ*_*r*_, it will be classified as positive, otherwise it is classed as negative.

In order to show the performance of different feature sets, we compared the proposed method not only with TranSparse, but also with cascade models with different feature sets. Results are shown in Table 2 (cascade-s, cascade-g, cascade-sg refer, respectively, to cascade models with only semantic features, only graph features and a concatenation of semantic and graph features).

**Table 2:** Link prediction results (accuracy).

Relation	TranSparse	Cascade-s	Cascade-g	Cascade-sg	Cascade model (proposed)
R1	0.9779	0.9690	0.9757	0.9779	**0.9801**
R2	0.6066	0.9322	**0.9615**	0.9528	0.9541
R3	0.9081	0.9205	0.8534	**0.9240**	0.9152
R4	0.8112	0.8913	0.9058	0.9203	**0.9275**
R5	0.7892	0.8722	**0.8927**	0.8666	0.8703
R6	0.9200	**0.9343**	0.8943	0.9257	0.9171
R7	0.9742	0.9836	0.9225	**0.9812**	0.9789
R8	0.9722	**1.0000**	0.9722	**0.9722**	0.9722
R9	0.7000	0.7500	**0.8167**	0.7333	0.7500
R10	0.9737	0.9770	0.9836	**0.9934**	0.9934
R11	0.9845	0.9845	0.9845	**0.9897**	**0.9897**
R12	0.7074	0.8353	**0.8823**	0.8419	0.8448
Avg.	0.8604	0.9208	0.9204	0.9233	**0.9299**

From the Table 2 we can observe that: (1) TranSparse does not perform well in the triplet classification task on Chem2Bio2Rdf, especially on relation bind (an extremely unbalanced relation); (2) cascade with only semantic features performs better than TranSparse in link prediction. Semantic features come from dissimilarity vectors of triplets in TranSparse. These features may be rough and inaccurate, but they can be fed into an additional classifier. Where Transparse merely counts elements, incorporation of addition classifier allows some weighting to be introduced, and so greatly improves link prediction. (3)The concatenation of semantic features and graph features does not significantly improve on only graph features in link prediction task. Cascade model with only graph features performs best in this task.

**Fig 6:**
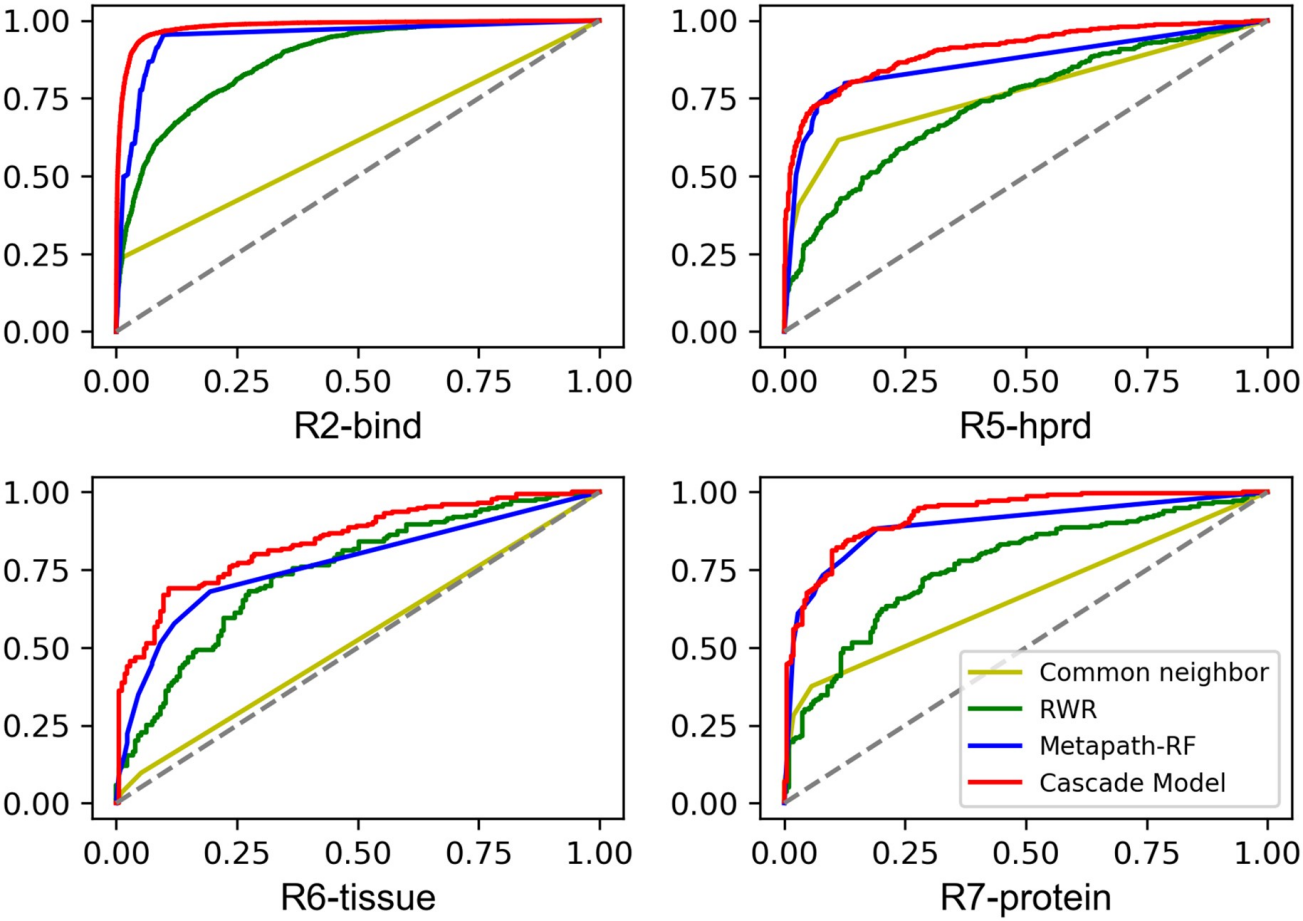
ROC Curve for part of relations on link prediction task.

We additionally evaluate the proposed method with some typical methods, such as popular heuristic score: common neighbor [38]; meta-path-based method: random forest on meta-path-based semantic features [13]; as well as RWR (random walk with restart) [39, 40]. The ROC curve (Fig 6) helps to illustrate the performance of the proposed models in the link prediction task on four relations: R2-*bind*, R5-*hprd*, R6-*tissue*, P7-*protein*. The cascade models significantly outperform the baselines on four selected relations: R2, R5, R6, R7. It shows the ability to obtain high precision results, making them useful for applying knowledge embedding to the biomedical domain. The cascade model is able to optimize the entity representation capability of TranSparse stage by stage and to improve its application value significantly. Common neighbor does not performance good because of the sparsity of data, the sparsity of data leads to zero common neighbor for most of entity pairs, but it seems better when comes to relation hprd, since the phenomenon of zero common neighbor in hprd relationship is less obvious. RWR takes diffusion features into consideration and achieves far better performance. The random forest, which contains meta-path-based semantics, is more effective. However, the proposed cascade model with both semantic features and topological features surpasses them with the best AUC without adding external domain knowledge.

We conducted further experiments to test the performance of different knowledge and graph embedding algorithms on the link prediction task. For graph embedding, we selected LINE and SDNE to calculate baselines, and used them instead of Node2vec in a different Cascade model. To better illustrate their performance in different situations, the performances were observed on 12 different relations (R1 to R12). Experimental results are shown in Table 3. The Cascade model with Node2vec performed better than the others overall, but not on every relation (eg, R6, R7 and R9). Cascade models showed an average of 11% absolute improvement compared to using only graph embedding for link prediction. Node2vec considers both local and global information, which is helpful for prediction but may result in bias on small-scale subgraphs. LINE preserves both first-order and second-order proximities, but more global topological information is neglected, so the performance is not as good as expected. SDNE obtains excellent performance on networks with highly non-linear attributes, such as Filckr, Youtube. While for biomedical network, the relations among entities are relatively stable, and Node2vec could be an optimal solution.

**Table 3:** Link Prediction based on settings of different graph embedding algorithms (accuracy).

Relation	Graph Embedding		Cascade-g			Cascade-sg			Cascade model (proposed)		
	L	N	L	S	N	L	S	N	L	S	N
R1	0.5000	0.9668	0.7323	0.7301	0.9757	0.9712	0.9690	0.9779	0.9735	0.9690	**0.9801**
R2	0.7218	0.9532	0.8466	0.6642	**0.9615**	0.9364	0.9344	0.9528	0.9363	0.9337	0.9541
R3	0.8834	0.7809	0.9028	0.6590	0.8534	0.9223	0.9205	**0.9240**	0.9205	0.9187	0.9205
R4	0.8841	0.8841	0.8696	0.7101	0.9058	0.8986	0.8841	0.9203	0.8986	0.8841	**0.9275**
R5	0.9263	0.8591	**0.9468**	0.6950	0.8927	0.8713	0.8722	0.8668	0.8685	0.8731	0.8703
R6	0.9000	0.7514	**0.9429**	0.6429	0.8943	0.9400	0.9314	0.9257	**0.9429**	0.9314	0.9171
R7	0.9390	0.9085	0.9577	0.6596	0.9225	**0.9836**	0.9836	0.9812	**0.9836**	**0.9836**	0.9789
R8	0.9167	0.9167	0.9444	0.5833	**0.9722**	0.9444	**1.0000**	0.9722	0.9444	**0.9722**	**0.9722**
R9	0.9333	0.7833	**0.9333**	0.7333	0.8167	0.8000	0.7500	0.7333	0.8000	0.7667	0.8100
R10	0.6546	0.9276	0.7566	0.7171	0.9836	0.9901	0.9770	**0.9934**	0.9901	0.9770	**0.9934**
R11	0.5000	0.9330	0.8041	0.7474	0.9845	0.9845	0.9845	**0.9897**	0.9845	0.9845	**0.9897**
R12	0.5000	0.8600	0.7423	0.6430	**0.8823**	0.8317	0.8369	0.8419	0.8346	0.8320	0.8448
Avg.	0.7716	0.8771	0.8650	0.6821	0.9204	0.9228	0.9203	0.9233	0.9231	0.9188	**0.9299**

L: LINE; S: SDNE; N: Node2vec

TranSparse is selected as knowledge embedding algorithms in the proposed cascade model.

TranSparse was replaced, variously, with TransE, TransH, and TransR as the knowledge embedding component of the proposed Cascade model, allowing us to generate a set of comparative results (see Table 4). The Cascade model with TransE performed best in the link prediction task. The performance of Cascade with TranSparse was ranked third; but the performance of all 4 models was close to each other. The percentage difference between averages of the lowest and best performing is just 0.63%. One reason for this consistency could be that the biochemical database used has a low number of link types: just 12 relations. FB15K, by comparison, has 961 relations. Another factor is that 70% of entity types in the biomedical data have only one relation (CHEBI, gene family, tissues and etc), creating an environment in which TransE performs well. Where there are many head and tail entities with multiple relations (such as Compound, gene and Disease), TransH, TransR and TranSparse perform better. For example, TranSparse outperformances the other baselines on R5: *hprd* (Gene-Gene), R8: *drug* (Disease-Compound). Though TranSparse has been shown to outperform the other baselines on link prediction tasks [22], the Cascade model can detect different relation patterns among entities, and strengthen its classification because of its stage by stage optimization. Another factor which may account for the similar performance of all four models is that, because there are many unconfirmed relationships among biomedical entities, we did not include entity pairs without relations in the testing dataset.

**Table 4:** Link Prediction based on settings of different knowledge embedding algorithms (accuracy).

Relation	Cascade (TransE)	Cascade (TransH)	Cascade (TransR)	Cascade (TranSparse)
R1	0.9823	**0.9889**	0.9757	0.9801
R2	**0.9604**	0.9593	0.954	0.9541
R3	**0.9329**	0.9276	0.9081	0.9205
R4	**0.9348**	**0.9348**	0.9203	0.9275
R5	**0.8713**	0.8573	0.8629	0.8703
R6	0.9171	**0.9200**	0.9000	0.9171
R7	0.9812	0.9765	**0.9836**	0.9789
R8	0.9444	0.9167	**0.9722**	**0.9722**
R9	0.8333	**0.8333**	**0.8333**	0.8100
R10	0.9934	0.9901	0.9868	**0.9934**
R11	0.9845	0.9794	**0.9897**	**0.9897**
R12	**0.9063**	0.9024	0.8488	0.8448
Avg.	**0.9368**	0.9322	0.9280	0.9299

Node2vec is selected as graph embedding algorithms in the proposed cascade model.

### Entity prediction

In the entity prediction task, two metrics are reported: mean rank and hits@10 rate, which represents overall performance and high-quality predictions respectively [19]. As described previously, test triplets were generated by removing head or tail entities from correct triplets and randomly replacing them with entities in the entity dictionary. The probability of all triplets was calculated and ranked in descending order. If a correct triplet is ranked in the top 10, then the count of hits@10 will be increased by one. Since some candidate triplets may already exist in the training set or test set, we filter candidate triplets during the ranking procedure, a process called *filtered setting* in TransE.

**Fig 7:**
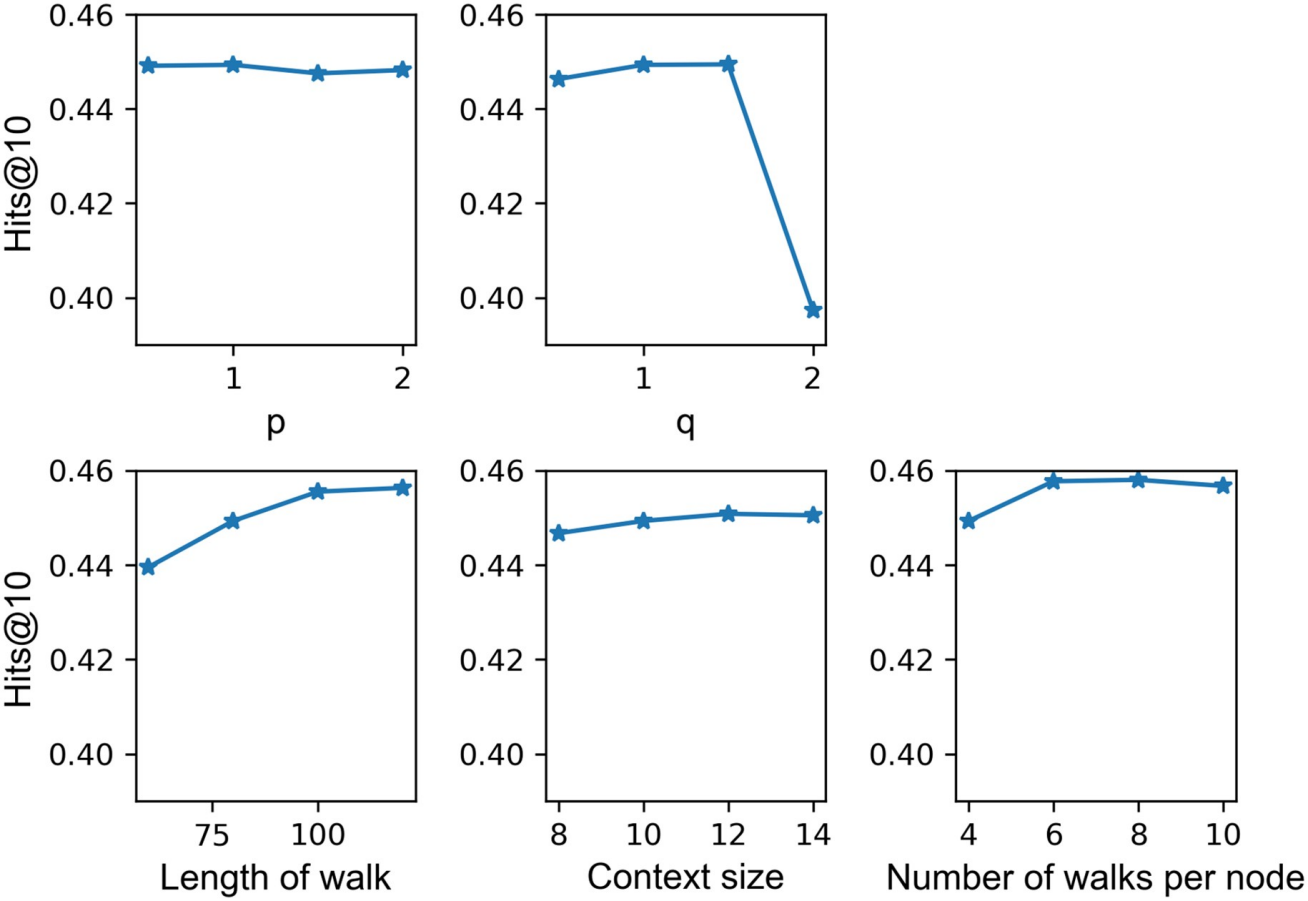
Sensitivity of proposed cascade model to parameters of Node2vec.

The graph features represented by the graph embedding model play an import role in the improvement of entity prediction from above. In order to figure out the effect of graph embedding, we also conducted some experiments with key parameters to show the parameter sensitivity (see Fig 7). Parameters p and q determine the combination of BFS and DFS sampling strategies. High p implies less likely to sample an already-visited node, and high q implies local view of underlying graph, namely, encouraging BFS behavior. It is apparent that the hits@10 rate is insensitive to parameter p, but collapsed when *q* > 1.5. This suggests that local structural features are import, but over reduction of global structural features can significantly impact the performance. Length of walk determines the number of sampling nodes; more walks would raise the hits@10 but eventually stabilize. Context size is the window size of sampled nodes sequence in training process, increasing window size would slightly impact the performance. Number of walks per node is the number of sampled node sequences for each node: longer walks per node mean more diversity, but also more complexity. As with the length of walk, a larger number of walks will raise the hits@10 rate up to a point, but once this point is exceeded, the performance declines.

We incorporate TranSparse and Node2vec into our proposed cascade model, and evaluate the performance of entity prediction on 12 relation types (R1 to R12). Typical knowledge and graph embedding algorithms are selected as baselines, besides, the settings of cascade-s, cascade-g, cascade-sg are the same as those in Table 2. Experimental results could be seen in Table 5. The mean rank of TranSparse is 3,772 (total 295,911 candidate entities) and the hits@10 is 23.98%. Compared to the original translation-base embedding model TransE, TranSparse has achieved great improvements, the mean rank drops from 10,120 to 3,772 and the hits@10 rises from 13.57% to 23.98%. The proposed cascade model achieves a significant improvement of 8.59% on hits@10 and 50% improvement on mean rank compared with TranSparse. Its hits@10 score has an average of 16.81% absolute improvement compared with typical knowledge and graph embedding algorithms (max: 28.8%, min: 8.59%). The performance of cascade models only incorporating knowledge embedding features cascade-s or graph embedding features cascade-g are not as significant as cascade-sg and proposed cascade model. Combining both knowledge embedding features and graph embedding distance features can make use of semantic information and graph topological information more efficient and make the proposed model more applicable for biomedical entities detection. The better performance of proposed cascade model compared to cascade-sg illustrates that graph embedding distance features are rough representation of entities and their relations, and they should be refined before concatenating operation.

**Table 5:** Entity prediction results.

Models	Mean Rank	hits@10(Avg.)
Node2Vec	4999	0.0377
TransE	10120	0.1357
TransH	3784	0.1571
TransR	4935	0.2176
TransSparse	3772	0.2398
Cascade-s	3678	0.2285
Cascade-g	3751	0.0700
Cascade-sg	2174	0.2988
Cascade model (proposed)	**1908**	**0.3257**

For the proposed cascade model, we selected the graph embedding algorithms LINE and SDNE to replace Node2vec as new baselines, and constructed a further experiment to test the performance of the revised model on the entity prediction task. Experimental results are shown in Table 6. As well as performing better at link prediction, the Cascade model with Node2vec proved better at entity prediction. It showed a 9.7% improvement over Cascade-LINE and a 9.6% improvement over Cascade-SDNE.

**Table 6:** Entity prediction based on settings of different graph embedding algorithms (hits@10).

Relation	(Predict head + Predict tail) / 2		
	Cascade-LINE	Cascade-SDNE	Cascade-Node2vec
R1	0.2677	0.2699	**0.4735**
R2	0.4798	0.4907	**0.5395**
R3	0.1414	0.1414	**0.2138**
R4	0.3841	0.4203	**0.5**
R5	0.1941	0.1885	**0.2146**
R6	0.1572	0.1629	**0.2**
R7	0.2888	0.2817	**0.4413**
R8	0.2500	0.3056	**0.4167**
R9	0.1833	0.1667	**0.2833**
R10	0.0625	0.0592	**0.0827**
R11	0.1289	0.1289	**0.2887**
R12	0.2022	0.1986	**0.2544**
Avg.	0.2283	0.2345	**0.3257**

TranSparse is selected as knowledge embedding algorithms in the proposed cascade model.

As with Table 6, we replaced TranSparse with TransE, TransR and TransH in the proposed model, and re-ran the entity prediction experiment (see Table 7). Overall the Cascade model with TranSparse performed best, with highest scores for Relation R1, R4, R5, R6, R8, R9, R10, R11 and R12. In overall performance, it improved by 3.3% on Cascade with TransE, 4.5% on Cascade with TransR, and 1.0% on Cascade with TransH. The 1st ranked entities in the entity prediction task needs more accurate representation of knowledge embedding than the Link Prediction task. This is especially true for 1-n. n-1, n-n situations, which suit TransH, TransR and TranSparse. For example, if Gene A and Gene B have the same Gene-family, TransE will tend to embed A and B close to each other, which limits detection of the Protein-Protein Interaction relationship. TranSparse, by contrast, generates a transformation matrix for each relation, and map the entity pairs into different relation-based space, which helps to resolve the problem.

**Table 7:** Entity prediction based on settings of different knowledge embedding algorithms (hits@10).

Relation	(Predict head + Predict tail) / 2			
	Cascade (TransE)	Cascade (TransH)	Cascade (TransR)	Cascade (TranSparse)
R1	0.4248	0.4647	0.4115	**0.4735**
R2	0.5449	0.5541	**0.5686**	0.5395
R3	0.1855	0.2226	0.1396	0.2138
R4	0.4203	0.4565	0.3551	**0.5000**
R5	0.1801	0.1903	0.1829	**0.2146**
R6	0.1743	0.18	0.1943	**0.2000**
R7	0.4179	**0.4625**	0.4062	0.4413
R8	0.3611	0.3889	0.3334	**0.4167**
R9	0.2334	0.25	0.2334	**0.2833**
R10	0.0724	0.0724	0.0823	**0.0827**
R11	0.2629	0.299	0.2629	**0.2887**
R12	0.2203	0.236	0.1976	**0.2544**
Avg.	0.2915	0.3148	0.2807	**0.3257**

Node2vec is selected as graph embedding algorithms in the proposed cascade model.

One main limitation of applying knowledge embedding methods in the biochemical domain is the imbalanced distribution of relations. We follow the discrimination of imbalance relation from TransE [19], the average number of heads h (respect. tails t) appearing in the data set, given a pair (r, t) (respect. a pair (h, r)), if the average number is larger than 1.5 then the argument n is labeled as N (stands for Many), and 1 otherwise. Experimental results are shown in Table 8, we can observe that nearly all the relations are N to N, and the performance of TranSparse on predicting N-side of entities is barely satisfactory. For example, bind is one of the most important relation in biochemical data set (over 70% relations belong to bind), it indicates a relation from *compound* (head) to gene (tail). The hits@10 of predicting compound is 12.22%, while the hits@10 of predicting gene is 85.6%. Since *bind* is an extreme imbalance relation, each gene may connect approximate 110 compounds through *bind* relation in average; hence it is difficult to encode all compounds to locate the same gene through relation bind by using knowledge embedding methods. The cascade model proposed in this paper introduces graph features to provide topological information for compounds encoding, and each encoded low-dimension vector contains more information to represent the corresponding compound, which is also helpful in finding the correct gene through bind relation. More detail improvements are shown in Table 8, proposed method outperforms TranSparse by 8.6% on average, with improvements on nearly all relations. For example, in the relationship bind, proposed method improves the hits@10 rate by 4.56% and 5.52% on predicting compound and gene respectively.

**Table 8:** Hit@10 rate in each relation on biochemical data set. n stands for the average number of head entities(respectively. tail entities) on dataset given a pair (r, t)(respectively (h,r)).

Index	Relation	n-n	Predict head		Predict tail	
			TranSparse	Cascade model	TranSparse	Cascade model
R1	Has chemical ontology	5.2-16	0.208	**0.469**	0.3319	**0.4779**
R2	Bind	109.4-2	0.1222	**0.1678**	0.856	**0.9112**
R3	Express	3.7-10.4	0.1413	**0.2261**	0.1307	**0.2014**
R4	Has gene family	1-21.6	0.6957	**0.8261**	0.1159	**0.1739**
R5	Protein-Protein interaction	4.4-4.1	0.1642	**0.194**	0.2071	**0.2351**
R6	Expressed in	2.5-19.2	0.2914	**0.32**	0.0457	**0.08**
R7	Has participants	2.8-55.1	0.4977	**0.6948**	0.1033	**0.1878**
R8	Treated by	1.6-4.8	0.5	**0.5556**	0.2222	**0.2778**
R9	Caused by	1.5-2.1	0.3333	**0.4333**	0	**0.1333**
R10	Induced by	11.2-8.4	0.0592	**0.0798**	0.0789	**0.0855**
R11	Has substructure	20.8-4.6	0.0309	**0.1134**	0.2165	**0.4639**
R12	Has gene ontology	9.1-6.1	0.14	**0.1788**	0.2630	**0.33**
Avg.	-	-	0.2653	**0.3549**	0.2143	**0.2965**

### Path prediction

In a set of experiments to measure the prediction performance and the applicability of the proposed method, we compute the probability of all potential compound-disease-gene paths, remove paths already existing in the dataset, and ranked the rest in descending order. Though partial relations along the reaction paths may already exist in the dataset (including training and test sets), it does not mean that the path is meaningless. For example, it may already be known that drug A can treat disease x, but the relevant mechanism or path is not known. The proposed method could suggest potentially relevant paths.

We calculated all possible compound-disease-gene paths and ranked them by probability. The top 100 paths are retained. The top 30 of these are listed in Table 9. Each path contains the relation treat, caused by, or bind between entities compound-disease, disease-gene, or compound-gene. For example, path Atazanavir-HIV-GAG-POL is detected by the proposed model. Compound Atazanavir can treat the disease HIV by binding the GAG-POL proteins. In this way, the properties of compounds can be predicted and associate them with specific disorders.

**Table 9:** Top 30 drug-disease-gene paths. The relations treat, caused by, and bind are associated with, respectively, drug-disease, disease-gene and drug-gene. The value x/y of indicates whether or not the relation exists in a data set: x = 1 indicates the presence of a relation in training or test sets; y = 1 indicates the presence of a relation in databases DisGeNET, DrugBank, etc.

Order number	Compound	Disease	Gene	Compound-Disease	Disease-Gene	Compound-Gene
1	Delavirdine	HIV	GAG-POL	1/1	0/1	1/0
2	Atazanavir	HIV	GAG-POL	1/1	0/1	0/0
3	Zidovudine	HIV	GAG-POL	1/1	0/1	1/0
4	Tenofovir disoproxil	HIV	GAG-POL	1/1	0/1	1/0
5	Zalcitabine	HIV	GAG-POL	1/1	0/1	1/0
6	Didanosine	HIV	GAG-POL	1/1	0/1	1/0
7	Emtricitabine	HIV	GAG-POL	1/1	0/1	1/0
8	Zidovudine	HIV	DEOA	1/1	0/0	0/0
9	Mercaptopurine	Leukemia	VDR	1/1	0/0	1/0
10	Efavirenz	HIV	GAG-POL	1/1	0/1	1/0
11	Zalcitabine	HIV	DEOA	1/1	0/0	0/0
12	Lamivudine	HIV	GAG-POL	1/1	0/1	1/0
13	Nevirapine	HIV	GAG-POL	1/1	0/1	1/0
14	Didanosine	HIV	DEOA	1/1	0/0	0/0
15	Emtricitabine	HIV	DEOA	1/1	0/0	0/0
16	Calcidiol	Hypoparathyroidism	VDR	1/1	0/0	1/1
17	Cidofovir	Immunodeficiency	UL54	1/1	0/0	1/0
18	Calcitriol	Hypocalcemia	CASR	1/1	1/0	0/0
19	Calcidiol	Hypocalcemia	CASR	1/1	1/0	0/0
20	Ganciclovir	Immunodeficiency	UL54	1/1	0/0	0/0
21	Daunorubicin	Leukemia	VDR	1/1	0/0	1/0
22	Stavudine	HIV	GAG-POL	1/1	0/1	1/0
23	Calcidiol	Hypoparathyroidism	CYP24A1	1/1	0/0	0/0
24	Vincristine	Leukemia	VDR	0/1	0/0	1/0
25	Calcitriol	Osteoporosis	FGF23	1/1	0/0	0/0
26	Risedronate	Osteoporosis	FDPS	1/1	0/0	1/0
27	Foscarnet	HIV	GAG-POL	0/1	0/1	0/0
28	Fluorouracil	Immunodeficiency	UNG	0/1	1/0	0/0
29	Propylthiouracil	HIV	DEOA	0/1	0/0	0/0
30	Lamivudine	HIV	DEOA	1/1	0/0	0/0

To validate predicted relations, they were matched with multiple databases. We matched relation treat (compound-disease) with DrugBank; caused by (disease-gene) with DisGeNET and UniProt; bind (compound-gene) with PubChem and dgidb. Results are shown in Table 10. In the path Atazanavir-HIV-GAG-POL, the relation treat between Atazanavir and HIV already exists in the dataset. The relation “caused by” between HIV and GAG-POL does not exist in the dataset, but is matched with the entry in the databases mentioned above, indicating a predictive relationship. The relation bind between Atazanavir and GAG-POL is neither in the dataset or the databases. This makes its status unknown but does not necessarily make it untrue: a literature review or further experimentation is needed to confirm its validity. Seventy-six relations between compounds and diseases are involved in the top 100 paths, but only 26 are unknown predictions requiring verification: 50 are already found in the dataset. The compound-disease prediction performed well, with all 26 compound-disease relations being matched in the databases. As for relation caused by (disease-gene), 1 out of 30 was confirmed with the databases whereas in the relation bind (compound-gene), no relations were confirmed, suggesting that the predicted paths are incorrect or need further investigation.

**Table 10:** Matching results of drug-disease-gene top 100 paths with database. “Number of triplets” is the number of triplets in specific relation involved in top 100 paths. “Predictions” is the number of relations neither in data sets nor in chosen databases. “Proven predictions” is the number of relations not in data sets but matched with chosen databases.

Relations	Triplets	Predictions	Proven predictions
Treat(compound-disease)	76	26	26
Caused by(disease-gene)	37	30	1
Bind(compound-target)	90	49	0

To further examine the unconfirmed paths, we conduct a literature analysis on the unsure predictions published in PubMed and PubMed Central, and some novel relations were found. For example, the proposed method predicted that VDR (vitamin D receptor) may be linked to causes of prostate_cancer. Low levels of vitamin D are implicated as a potential risk factor for prostate cancer. Some research suggests that the vitamin D receptor (VDR) gene is important in the onset and progression of prostate cancer and VDR gene polymorphisms might be associated with prostate cancer risk [41, 42].

We also found some novel interaction paths. For example, in the path of Calcitriol-Osteoporosis-FGF23, calcitriol is known to be useful in the treatment of osteoporosis, but it is not known why. The proposed method infers that calcitriol can bind FGF23, which may be one of the causes of osteoporosis. This is supported by literature analysis, where research suggest that FGF23 overexpression suppresses not only osteoblast differentiation but also matrix mineralization [43]. Serum FGF-23 level is a significant determinant of increased bone turnover at early periods in postmenopausal osteoporosis patients [44] so compounds which target FGF23 might be of value in the treatment of osteoporosis. Georgiadou et al [45] found that administration of calcitriol and sevelamer in combination restrains the increase of FGF23, indicating that calcitriol’s role in treating osteoporosis may be due to the binding of FGF23.

Another interesting prediction to emerge is a relation between Gemcitabine and Lymphoma-UNG. There is no relation between them in the dataset but the result predicts that gemcitabine may help to prevent the development of lymphoma by restraining the expression of UNG. B cell proliferation depends on the protective repair by uracil-DNA glycosylase (UNG). UNG deficiency blocks the proliferation of tumor B cells expressing AID (Activation-induced deaminase), which deaminates nonimmunoglobulin genes, may cause B cell lymphoma if failure to faithfully repair these off-target lesions [46]. Thus, targeting this gene may help with the treatment of certain types of lymphoma. Gemcitabine is predicted to bind UNG and treat lymphoma from the proposed method. It is already known that Gemcitabine can treat lymphoma to some extent, the question of whether Gemcitabine bind UNG or not has yet to be explored.

## Conclusion

This paper have proposed a cascade model for biochemical link predictions. The novelty of the model lies in the combination of semantic features and graph features in a multi-relational biochemical data set in which the semantic features derive from the translation-based knowledge embedding model and the graph features are learned from the mainstream graph embedding model Node2vec. The translation-based knowledge embedding model is efficient and scalable and the embedding vectors from Node2vec encode local and global topological information. The cascade learning model uses series functions to relocate the triplets in the feature space and achieves noticeable improvements.

Three tasks have been set in this paper, namely link prediction, entity prediction, and path prediction. The evaluation metrics show promising results in both link prediction and entity prediction. In path prediction, we combine meta-paths to predict the potential reaction paths. Predictions have been matched to authoritative databases such as Drug Bank and PubChem, and explored in the light of published evidence, which suggested that some of them offered promising insights which could be of value in drug discovery or drug repositioning. For example, from drug-disease-gene path, we were able to identify the possibility that some known drugs and some target genes may have associations with certain specific diseases.

It should be emphasized that the proposed cascade learning framework could also incorporate domain unique features, such as domain knowledge-based compound embedding, gene embedding, etc. This could further improve its performance; and because the knowledge embedding model is intended for multi-relational data, predictions are not limited to one relation. Although this paper is mainly concerned with Drug-Target interaction (DTI), its findings can be adapted for other purposes, such as predicting protein-protein interactions (PPI) and identifying associations between drugs and adverse side effects. There is scope for the development of an integrated model which can learn from both semantic and graph features. Future researchers can focus on one relation and can add more contexts to refine predictions, for example, drug similarity, gene similarity.

## Supporting information

S1 Data Experiment data. The data used in the experiment, also can be found at https://github.com/lxm36/Cascade-Embedding.
(ZIP)
